# 2-Methyl-4-(4-methyl­piperazin-1-yl)-10*H*-thieno[2,3-*b*][1,5]benzodiazepine (olanzapine) propan-2-ol disolvate

**DOI:** 10.1107/S1600536813009811

**Published:** 2013-04-20

**Authors:** Rajni M. Bhardwaj, Alastair J. Florence

**Affiliations:** a Strathclyde Institute of Pharmacy and Biomedical Sciences, University of Strathclyde, 161 Cathedral Street, Glasgow G4 0RE, Scotland

## Abstract

In the title solvate, C_17_H_20_N_4_S·2C_3_H_8_O, pairs of olanzapine mol­ecules related by a centre of inversion stack along the *a* axis, forming columns, which are packed parallel to each other along the *b* axis, forming a sheet arrangement. The columns within these sheets are hydrogen bonded to each other through the propan-2-ol solvent mol­ecules. The diazepine ring of the olanzapine exists in a puckered conformation with the thiophene and phenyl rings making a dihedral angle of 57.66 (7)° and the piperazine ring adopts a chair conformation with the methyl group in an equatorial position.

## Related literature

For literature on olanzapine and related structural studies, see: Fulton & Goa (1997 ▶); Sanger *et al.* (2001 ▶); Tollefson *et al.* (1997 ▶); Reutzel-Edens *et al.* (2003 ▶); Bhardwaj *et al.* (2013 ▶). For details of experimental methods used, see: Florence *et al.* (2003 ▶). For details of *XPac*, see: Gelbrich & Hursthouse (2005 ▶).
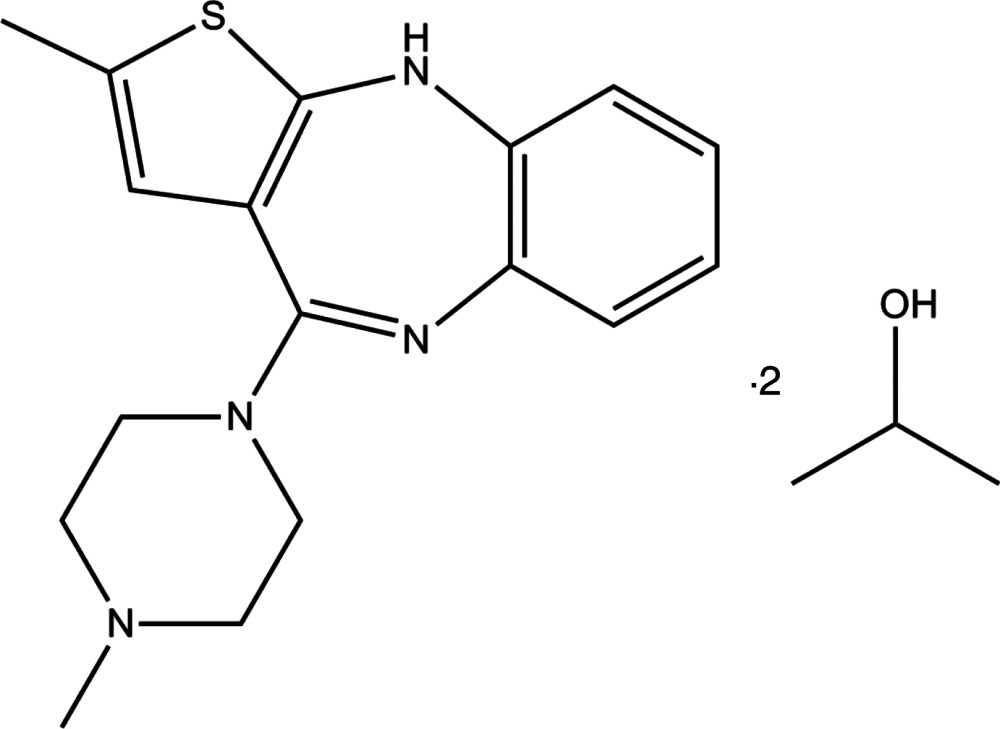


## Experimental

### 

#### Crystal data

C_17_H_20_N_4_S·2C_3_H_8_O
*M* *_r_* = 432.62
Triclinic, 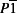

*a* = 9.9621 (5) Å
*b* = 10.8702 (6) Å
*c* = 12.2298 (7) Å
α = 70.421 (2)°
β = 74.560 (2)°
γ = 77.296 (2)°
*V* = 1190.24 (11) Å^3^
*Z* = 2
Mo *K*α radiation
μ = 0.16 mm^−1^
*T* = 123 K
0.41 × 0.34 × 0.11 mm

#### Data collection

Bruker APEXII CCD diffractometer
Absorption correction: multi-scan (*SADABS*; Bruker, 2007 ▶) *T*_min_ = 0.581, *T*_max_ = 0.745
16365 measured reflections
4880 independent reflections
4073 reflections with *I* > 2σ(*I*)
*R*_int_ = 0.025

#### Refinement

*R*[*F* ^2^ > 2σ(*F* ^2^)] = 0.038
*wR*(*F* ^2^) = 0.106
*S* = 1.04
4880 reflections
289 parameters
H atoms treated by a mixture of independent and constrained refinement
Δρ_max_ = 0.33 e Å^−3^
Δρ_min_ = −0.29 e Å^−3^

### 

Data collection: *APEX2* (Bruker, 2007 ▶); cell refinement: *SAINT* (Bruker, 2007 ▶); data reduction: *SAINT*; program(s) used to solve structure: *SHELXS97* (Sheldrick, 2008 ▶) and *WinGX* (Farrugia, 2012 ▶); program(s) used to refine structure: *SHELXL97* (Sheldrick, 2008 ▶) and *WinGX* (Farrugia, 2012 ▶); molecular graphics: *Mercury* (Macrae *et al.*, 2008 ▶) and *ORTEP-3 for Windows* (Farrugia, 2012 ▶); software used to prepare material for publication: *enCIFer* (Allen *et al.*, 2004 ▶) and *publCIF* (Westrip, 2010 ▶).

## Supplementary Material

Crystal structure: contains datablock(s) I, global. DOI: 10.1107/S1600536813009811/fj2624sup1.cif

Structure factors: contains datablock(s) I. DOI: 10.1107/S1600536813009811/fj2624Isup2.hkl

Supplementary material file. DOI: 10.1107/S1600536813009811/fj2624Isup3.cml

Additional supplementary materials:  crystallographic information; 3D view; checkCIF report

## Figures and Tables

**Table 1:** Hydrogen-bond geometry (Å, °)

*D*—H⋯*A*	*D*—H	H⋯*A*	*D*⋯*A*	*D*—H⋯*A*
N1—H1*N*⋯O2*S* ^i^	0.90 (2)	2.04 (2)	2.933 (2)	177 (2)
O1*S*—H3*S*⋯N4^ii^	0.86 (2)	1.94 (2)	2.778 (2)	167 (2)

Symmetry codes: (i) 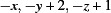; (ii) 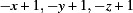.

**Fig. 1.**
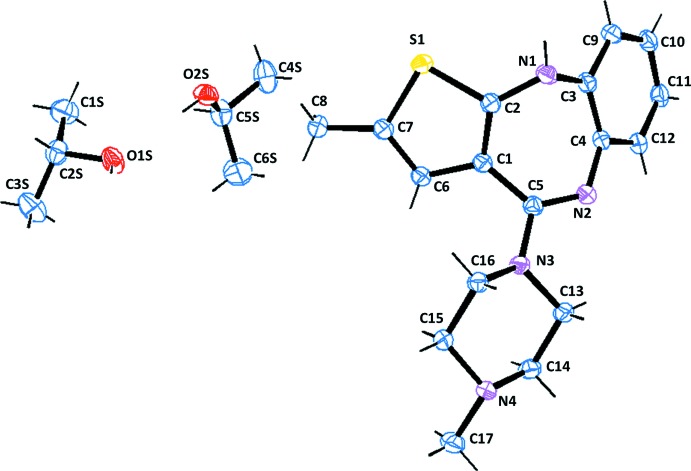
The asymmetric unit of olanzapine propan-2-ol solvate. Displacement ellipsoids are drawn at 50% probability level.

**Fig. 2.**
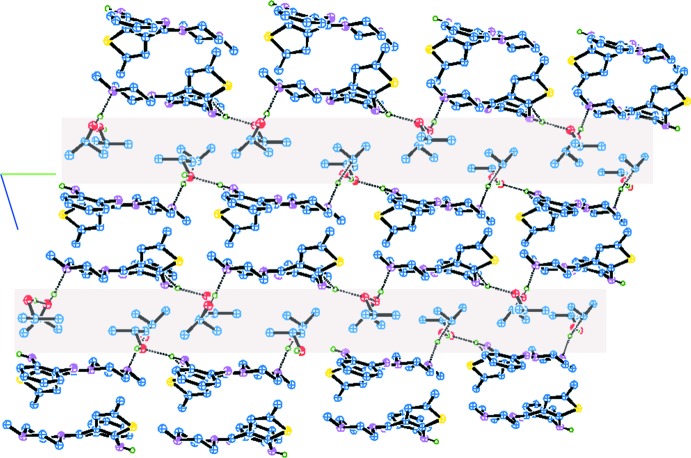
The crystal packing in olanzapine propan-2-ol solvate, viewed down the a-axis. H-bonds are shown by black dotted line. Carbon, nitrogen, oxygen, sulfur and hydrogen atoms are shown in blue, violet, red, yellow and green colour respectively. Other hydrogen atoms are omitted for clarity.

## supplementary crystallographic information

### Comment

2-methyl-4-(4-methylpiperazin-1-yl)-10*H*-thieno[2,3-*b*][1,5]benzodiazepine (Olanzapine, OZPN) is used in the treatment of schizophrenia and related psychoses (Fulton *et al.*, 1997; Tollefson *et al.*, 1997; Sanger *et al.*, 2001). The compound is known to exist in three anhydrous polymorphic forms and 56 solvates including four hydrates have been reported. The crystal structures of two polymorphs and 33 solvates have been reported and all are based on a centrosymmetric dimer motif, which is considered to be the structural building block (Reutzel-Edens *et al.*, 2003; Bhardwaj *et al.*, 2013). The sample of OZPN propan-2-ol solvate was isolated during an experimental physical form screen. The sample was identified as a novel form using multi-sample foil transmission X-ray powder diffraction analysis (Florence *et al.*, 2003).

A suitable sample for single-crystal X-ray diffraction analysis was obtained from slow evaporation of saturated propan-2-ol solution at room temperature. The title compound crystallizes in space group *P*-1 with one molecule of OZPN and two molecules of propan-2-ol in the asymmetric unit (Fig. 1). OZPN molecules form centrosymmetric dimers, which stack along the *a*-direction to form columns. These columns further stack along the *b*-direction to form sheets and are H-bonded to each other through propan-2-ol molecules, which are present between the sheets. The solvent separated sheets stack along the *c*-direction to form three-dimensional structure (Fig. 2). XPac (Gelbrich *et al.*, 2005) analysis revealed that this solvate shares 2-D similarity with form I (CSD refcode: UNOGIN01) and dihydrate D (CSD refcode: AQOMAU). The major difference between the structures arises from the relative orientation of the sheets due to incorporation of solvent molecules between them.

### Experimental

A single plate shaped crystal was grown from the saturated solution of OZPN in propan-2-ol by isothermal solvent evaporation at 298 K.

### Refinement

The positions of the nitrogen and oxygen-bound H atoms were refined freely. All other H atoms were placed in calculated positions and refined in riding modes with C—H = 0.95, 0.98 and 0.99 Å for the aromatic CH, CH_3_ and CH_2_ groups respectively. The *U*_iso_(H) values were set to 1.2 and 1.5 times *U*_eq_ of their parent C atoms for the aromatic CH, CH_2_ and CH_3_ groups respectively.

### Crystal data

**Table:**

C_17_H_20_N_4_S·2C_3_H_8_O	*Z* = 2
*M**_r_* = 432.62	*F*(000) = 468
Triclinic, *P*1	*D*_x_ = 1.207 Mg m^−^^3^
Hall symbol: -P 1	Mo *K*α radiation, λ = 0.71073 Å
*a* = 9.9621 (5) Å	Cell parameters from 8504 reflections
*b* = 10.8702 (6) Å	θ = 2.3–26.5°
*c* = 12.2298 (7) Å	µ = 0.16 mm^−^^1^
α = 70.421 (2)°	*T* = 123 K
β = 74.560 (2)°	Plate, yellow
γ = 77.296 (2)°	0.41 × 0.34 × 0.11 mm
*V* = 1190.24 (11) Å^3^	

### Data collection

**Table:**

Bruker APEXII CCD diffractometer	4880 independent reflections
Radiation source: fine-focus sealed tube	4073 reflections with *I* > 2σ(*I*)
Graphite monochromator	*R*_int_ = 0.025
φ and ω scans	θ_max_ = 26.6°, θ_min_ = 1.8°
Absorption correction: multi-scan (*SADABS*; Bruker, 2007)	*h* = −12→10
*T*_min_ = 0.581, *T*_max_ = 0.745	*k* = −11→13
16365 measured reflections	*l* = −15→15

### Refinement

**Table:**

Refinement on *F*^2^	Primary atom site location: structure-invariant direct methods
Least-squares matrix: full	Secondary atom site location: difference Fourier map
*R*[*F*^2^ > 2σ(*F*^2^)] = 0.038	Hydrogen site location: inferred from neighbouring sites
*wR*(*F*^2^) = 0.106	H atoms treated by a mixture of independent and constrained refinement
*S* = 1.04	*w* = 1/[σ^2^(*F*_o_^2^) + (0.055*P*)^2^ + 0.4568*P*] where *P* = (*F*_o_^2^ + 2*F*_c_^2^)/3
4880 reflections	(Δ/σ)_max_ < 0.001
289 parameters	Δρ_max_ = 0.33 e Å^−^^3^
0 restraints	Δρ_min_ = −0.29 e Å^−^^3^

### Special details

**Table:**

Geometry. All s.u.'s (except the s.u. in the dihedral angle between two l.s. planes) are estimated using the full covariance matrix. The cell s.u.'s are taken into account individually in the estimation of s.u.'s in distances, angles and torsion angles; correlations between s.u.'s in cell parameters are only used when they are defined by crystal symmetry. An approximate (isotropic) treatment of cell s.u.'s is used for estimating s.u.'s involving l.s. planes.
Refinement. Refinement of *F*^2^ against ALL reflections. The weighted *R*-factor *wR* and goodness of fit *S* are based on *F*^2^, conventional *R*-factors *R* are based on *F*, with *F* set to zero for negative *F*^2^. The threshold expression of *F*^2^ > 2σ(*F*^2^) is used only for calculating *R*-factors(gt) *etc*. and is not relevant to the choice of reflections for refinement. *R*-factors based on *F*^2^ are statistically about twice as large as those based on *F*, and *R*- factors based on ALL data will be even larger.

### Fractional atomic coordinates and isotropic or equivalent isotropic displacement parameters (Å^2^)

**Table:**

	*x*	*y*	*z*	*U*_iso_*/*U*_eq_	
H1N	−0.1193 (19)	0.9466 (19)	0.1993 (16)	0.032 (5)*	
H3S	0.514 (2)	0.758 (2)	0.825 (2)	0.050 (6)*	
H1S	0.260 (3)	0.830 (2)	0.854 (2)	0.058 (7)*	
S1	0.04115 (4)	0.91878 (4)	0.36298 (3)	0.02407 (11)	
N1	−0.09642 (13)	0.85854 (13)	0.22328 (11)	0.0230 (3)	
N4	0.36303 (13)	0.26018 (12)	0.32261 (11)	0.0219 (3)	
N3	0.14149 (12)	0.47811 (12)	0.30511 (11)	0.0217 (3)	
N2	−0.08278 (13)	0.56928 (12)	0.28169 (11)	0.0219 (3)	
C14	0.21368 (16)	0.24248 (14)	0.35573 (14)	0.0244 (3)	
H14A	0.1787	0.2325	0.4415	0.029*	
H14B	0.2046	0.1606	0.3414	0.029*	
C16	0.28887 (15)	0.49962 (14)	0.26562 (14)	0.0223 (3)	
H16A	0.2985	0.5831	0.2769	0.027*	
H16B	0.3210	0.5068	0.1801	0.027*	
C5	0.03110 (15)	0.57973 (14)	0.30703 (12)	0.0200 (3)	
C6	0.13109 (15)	0.67258 (14)	0.43396 (13)	0.0213 (3)	
H6	0.1755	0.5898	0.4756	0.026*	
C7	0.13712 (15)	0.78773 (15)	0.45145 (13)	0.0228 (3)	
C13	0.12395 (16)	0.35797 (14)	0.28589 (14)	0.0229 (3)	
H13A	0.1534	0.3649	0.2003	0.027*	
H13B	0.0238	0.3448	0.3126	0.027*	
C12	−0.33300 (16)	0.60148 (15)	0.33644 (13)	0.0246 (3)	
H12	−0.3275	0.5082	0.3590	0.030*	
C3	−0.21953 (15)	0.79562 (14)	0.26365 (12)	0.0211 (3)	
C15	0.37834 (16)	0.38554 (15)	0.33649 (14)	0.0243 (3)	
H15A	0.4783	0.3993	0.3090	0.029*	
H15B	0.3493	0.3817	0.4214	0.029*	
C2	−0.00519 (15)	0.81452 (14)	0.30203 (13)	0.0208 (3)	
C10	−0.47383 (16)	0.81431 (16)	0.30737 (14)	0.0259 (3)	
H10	−0.5633	0.8678	0.3091	0.031*	
C4	−0.20831 (15)	0.65708 (14)	0.29640 (13)	0.0213 (3)	
C8	0.20862 (17)	0.81074 (16)	0.53509 (15)	0.0273 (3)	
H8A	0.1404	0.8144	0.6087	0.041*	
H8B	0.2467	0.8944	0.4981	0.041*	
H8C	0.2854	0.7384	0.5530	0.041*	
C17	0.44267 (18)	0.14983 (16)	0.39751 (15)	0.0301 (4)	
H17A	0.5423	0.1610	0.3746	0.045*	
H17B	0.4325	0.0669	0.3873	0.045*	
H17C	0.4066	0.1476	0.4808	0.045*	
C11	−0.46419 (16)	0.67801 (16)	0.34425 (14)	0.0259 (3)	
H11	−0.5471	0.6373	0.3747	0.031*	
C1	0.05185 (15)	0.68643 (14)	0.34722 (13)	0.0206 (3)	
C9	−0.35177 (16)	0.87206 (15)	0.26790 (13)	0.0241 (3)	
H9	−0.3585	0.9655	0.2433	0.029*	
O1S	0.44594 (13)	0.77562 (13)	0.87986 (11)	0.0348 (3)	
C2S	0.50035 (18)	0.80746 (17)	0.96257 (14)	0.0318 (4)	
H2S	0.5619	0.8773	0.9179	0.038*	
C3S	0.58641 (19)	0.6878 (2)	1.02927 (16)	0.0425 (5)	
H3S1	0.5278	0.6176	1.0712	0.064*	
H3S2	0.6211	0.7107	1.0868	0.064*	
H3S3	0.6664	0.6572	0.9733	0.064*	
C1S	0.3754 (2)	0.8628 (2)	1.04315 (16)	0.0420 (5)	
H1S1	0.3228	0.9397	0.9952	0.063*	
H1S2	0.4087	0.8892	1.0994	0.063*	
H1S3	0.3140	0.7952	1.0869	0.063*	
O2S	0.17158 (13)	0.85357 (11)	0.84879 (10)	0.0308 (3)	
C5S	0.08774 (19)	0.77005 (18)	0.94873 (15)	0.0345 (4)	
H6S	0.1012	0.7803	1.0232	0.041*	
C4S	−0.0636 (2)	0.8177 (2)	0.93962 (18)	0.0464 (5)	
H5S1	−0.0883	0.9095	0.9413	0.070*	
H5S2	−0.1242	0.7625	1.0066	0.070*	
H5S3	−0.0771	0.8120	0.8650	0.070*	
C6S	0.1325 (3)	0.62811 (19)	0.9508 (2)	0.0577 (6)	
H7S1	0.1192	0.6169	0.8783	0.087*	
H7S2	0.0755	0.5723	1.0202	0.087*	
H7S3	0.2320	0.6026	0.9552	0.087*	

### Atomic displacement parameters (Å^2^)

**Table:**

	*U*^11^	*U*^22^	*U*^33^	*U*^12^	*U*^13^	*U*^23^
S1	0.0235 (2)	0.01788 (19)	0.0328 (2)	−0.00162 (14)	−0.00854 (15)	−0.00866 (15)
N1	0.0219 (6)	0.0188 (6)	0.0268 (6)	−0.0008 (5)	−0.0093 (5)	−0.0029 (5)
N4	0.0211 (6)	0.0199 (6)	0.0267 (6)	0.0027 (5)	−0.0098 (5)	−0.0092 (5)
N3	0.0173 (6)	0.0179 (6)	0.0316 (7)	−0.0013 (5)	−0.0065 (5)	−0.0092 (5)
N2	0.0208 (6)	0.0206 (6)	0.0259 (6)	−0.0006 (5)	−0.0085 (5)	−0.0074 (5)
C14	0.0241 (8)	0.0187 (7)	0.0306 (8)	−0.0019 (6)	−0.0050 (6)	−0.0088 (6)
C16	0.0181 (7)	0.0198 (7)	0.0306 (8)	−0.0027 (6)	−0.0051 (6)	−0.0096 (6)
C5	0.0203 (7)	0.0181 (7)	0.0208 (7)	−0.0029 (6)	−0.0051 (6)	−0.0039 (5)
C6	0.0182 (7)	0.0204 (7)	0.0258 (7)	−0.0008 (6)	−0.0063 (6)	−0.0072 (6)
C7	0.0188 (7)	0.0232 (7)	0.0272 (7)	−0.0017 (6)	−0.0054 (6)	−0.0088 (6)
C13	0.0202 (7)	0.0201 (7)	0.0310 (8)	−0.0023 (6)	−0.0068 (6)	−0.0103 (6)
C12	0.0247 (8)	0.0246 (8)	0.0283 (8)	−0.0023 (6)	−0.0121 (6)	−0.0082 (6)
C3	0.0210 (7)	0.0236 (7)	0.0201 (7)	−0.0008 (6)	−0.0074 (6)	−0.0071 (6)
C15	0.0210 (7)	0.0249 (8)	0.0315 (8)	0.0009 (6)	−0.0098 (6)	−0.0135 (6)
C2	0.0171 (7)	0.0209 (7)	0.0253 (7)	−0.0022 (6)	−0.0044 (6)	−0.0084 (6)
C10	0.0201 (7)	0.0337 (9)	0.0267 (8)	0.0042 (6)	−0.0096 (6)	−0.0140 (7)
C4	0.0211 (7)	0.0231 (7)	0.0223 (7)	0.0005 (6)	−0.0099 (6)	−0.0082 (6)
C8	0.0261 (8)	0.0267 (8)	0.0346 (8)	−0.0025 (6)	−0.0105 (7)	−0.0138 (7)
C17	0.0330 (9)	0.0266 (8)	0.0309 (8)	0.0065 (7)	−0.0143 (7)	−0.0094 (7)
C11	0.0201 (7)	0.0337 (8)	0.0279 (8)	−0.0037 (6)	−0.0083 (6)	−0.0119 (6)
C1	0.0167 (7)	0.0201 (7)	0.0256 (7)	−0.0008 (6)	−0.0050 (6)	−0.0084 (6)
C9	0.0259 (8)	0.0225 (7)	0.0244 (7)	0.0027 (6)	−0.0100 (6)	−0.0079 (6)
O1S	0.0261 (6)	0.0516 (8)	0.0322 (6)	0.0042 (5)	−0.0095 (5)	−0.0228 (6)
C2S	0.0355 (9)	0.0347 (9)	0.0283 (8)	−0.0080 (7)	−0.0084 (7)	−0.0104 (7)
C3S	0.0288 (9)	0.0608 (13)	0.0309 (9)	0.0016 (9)	−0.0060 (7)	−0.0098 (9)
C1S	0.0504 (12)	0.0442 (11)	0.0350 (10)	0.0068 (9)	−0.0151 (9)	−0.0201 (8)
O2S	0.0258 (6)	0.0242 (6)	0.0362 (6)	−0.0032 (5)	−0.0058 (5)	−0.0019 (5)
C5S	0.0340 (9)	0.0412 (10)	0.0281 (8)	−0.0136 (8)	−0.0074 (7)	−0.0042 (7)
C4S	0.0314 (10)	0.0684 (14)	0.0401 (10)	−0.0170 (9)	−0.0035 (8)	−0.0139 (10)
C6S	0.0651 (15)	0.0318 (10)	0.0726 (15)	−0.0172 (10)	−0.0297 (12)	0.0074 (10)

### Geometric parameters (Å, º)

**Table:**

S1—C7	1.7415 (15)	C10—C11	1.386 (2)
S1—C2	1.7415 (15)	C10—C9	1.388 (2)
N1—C2	1.3959 (19)	C10—H10	0.9500
N1—C3	1.4268 (19)	C8—H8A	0.9800
N1—H1N	0.897 (19)	C8—H8B	0.9800
N4—C17	1.4627 (19)	C8—H8C	0.9800
N4—C14	1.4723 (19)	C17—H17A	0.9800
N4—C15	1.4728 (18)	C17—H17B	0.9800
N3—C5	1.3782 (18)	C17—H17C	0.9800
N3—C13	1.4558 (18)	C11—H11	0.9500
N3—C16	1.4633 (18)	C9—H9	0.9500
N2—C5	1.2897 (19)	O1S—C2S	1.430 (2)
N2—C4	1.4061 (18)	O1S—H3S	0.86 (2)
C14—C13	1.516 (2)	C2S—C3S	1.504 (2)
C14—H14A	0.9900	C2S—C1S	1.515 (2)
C14—H14B	0.9900	C2S—H2S	1.0000
C16—C15	1.513 (2)	C3S—H3S1	0.9800
C16—H16A	0.9900	C3S—H3S2	0.9800
C16—H16B	0.9900	C3S—H3S3	0.9800
C5—C1	1.474 (2)	C1S—H1S1	0.9800
C6—C7	1.355 (2)	C1S—H1S2	0.9800
C6—C1	1.434 (2)	C1S—H1S3	0.9800
C6—H6	0.9500	O2S—C5S	1.436 (2)
C7—C8	1.501 (2)	O2S—H1S	0.87 (2)
C13—H13A	0.9900	C5S—C6S	1.502 (3)
C13—H13B	0.9900	C5S—C4S	1.504 (3)
C12—C11	1.385 (2)	C5S—H6S	1.0000
C12—C4	1.399 (2)	C4S—H5S1	0.9800
C12—H12	0.9500	C4S—H5S2	0.9800
C3—C9	1.393 (2)	C4S—H5S3	0.9800
C3—C4	1.409 (2)	C6S—H7S1	0.9800
C15—H15A	0.9900	C6S—H7S2	0.9800
C15—H15B	0.9900	C6S—H7S3	0.9800
C2—C1	1.368 (2)		
			
C7—S1—C2	91.98 (7)	C7—C8—H8A	109.5
C2—N1—C3	114.13 (12)	C7—C8—H8B	109.5
C2—N1—H1N	113.8 (12)	H8A—C8—H8B	109.5
C3—N1—H1N	110.7 (12)	C7—C8—H8C	109.5
C17—N4—C14	109.50 (12)	H8A—C8—H8C	109.5
C17—N4—C15	110.02 (12)	H8B—C8—H8C	109.5
C14—N4—C15	110.22 (11)	N4—C17—H17A	109.5
C5—N3—C13	120.29 (12)	N4—C17—H17B	109.5
C5—N3—C16	122.85 (12)	H17A—C17—H17B	109.5
C13—N3—C16	110.77 (11)	N4—C17—H17C	109.5
C5—N2—C4	123.11 (13)	H17A—C17—H17C	109.5
N4—C14—C13	112.02 (12)	H17B—C17—H17C	109.5
N4—C14—H14A	109.2	C12—C11—C10	119.60 (14)
C13—C14—H14A	109.2	C12—C11—H11	120.2
N4—C14—H14B	109.2	C10—C11—H11	120.2
C13—C14—H14B	109.2	C2—C1—C6	112.09 (13)
H14A—C14—H14B	107.9	C2—C1—C5	121.52 (13)
N3—C16—C15	109.49 (12)	C6—C1—C5	126.38 (13)
N3—C16—H16A	109.8	C10—C9—C3	121.24 (14)
C15—C16—H16A	109.8	C10—C9—H9	119.4
N3—C16—H16B	109.8	C3—C9—H9	119.4
C15—C16—H16B	109.8	C2S—O1S—H3S	109.0 (15)
H16A—C16—H16B	108.2	O1S—C2S—C3S	110.46 (14)
N2—C5—N3	118.01 (13)	O1S—C2S—C1S	106.97 (14)
N2—C5—C1	126.03 (13)	C3S—C2S—C1S	112.88 (15)
N3—C5—C1	115.78 (12)	O1S—C2S—H2S	108.8
C7—C6—C1	114.22 (13)	C3S—C2S—H2S	108.8
C7—C6—H6	122.9	C1S—C2S—H2S	108.8
C1—C6—H6	122.9	C2S—C3S—H3S1	109.5
C6—C7—C8	128.92 (14)	C2S—C3S—H3S2	109.5
C6—C7—S1	110.50 (11)	H3S1—C3S—H3S2	109.5
C8—C7—S1	120.55 (11)	C2S—C3S—H3S3	109.5
N3—C13—C14	108.59 (12)	H3S1—C3S—H3S3	109.5
N3—C13—H13A	110.0	H3S2—C3S—H3S3	109.5
C14—C13—H13A	110.0	C2S—C1S—H1S1	109.5
N3—C13—H13B	110.0	C2S—C1S—H1S2	109.5
C14—C13—H13B	110.0	H1S1—C1S—H1S2	109.5
H13A—C13—H13B	108.4	C2S—C1S—H1S3	109.5
C11—C12—C4	122.09 (14)	H1S1—C1S—H1S3	109.5
C11—C12—H12	119.0	H1S2—C1S—H1S3	109.5
C4—C12—H12	119.0	C5S—O2S—H1S	108.9 (15)
C9—C3—C4	119.78 (14)	O2S—C5S—C6S	110.43 (16)
C9—C3—N1	119.63 (13)	O2S—C5S—C4S	107.23 (14)
C4—C3—N1	120.57 (13)	C6S—C5S—C4S	112.72 (17)
N4—C15—C16	110.50 (12)	O2S—C5S—H6S	108.8
N4—C15—H15A	109.5	C6S—C5S—H6S	108.8
C16—C15—H15A	109.5	C4S—C5S—H6S	108.8
N4—C15—H15B	109.5	C5S—C4S—H5S1	109.5
C16—C15—H15B	109.5	C5S—C4S—H5S2	109.5
H15A—C15—H15B	108.1	H5S1—C4S—H5S2	109.5
C1—C2—N1	125.87 (13)	C5S—C4S—H5S3	109.5
C1—C2—S1	111.18 (11)	H5S1—C4S—H5S3	109.5
N1—C2—S1	122.83 (11)	H5S2—C4S—H5S3	109.5
C11—C10—C9	119.46 (14)	C5S—C6S—H7S1	109.5
C11—C10—H10	120.3	C5S—C6S—H7S2	109.5
C9—C10—H10	120.3	H7S1—C6S—H7S2	109.5
C12—C4—N2	116.18 (13)	C5S—C6S—H7S3	109.5
C12—C4—C3	117.75 (13)	H7S1—C6S—H7S3	109.5
N2—C4—C3	125.76 (13)	H7S2—C6S—H7S3	109.5
			
C17—N4—C14—C13	176.70 (12)	C7—S1—C2—N1	175.64 (13)
C15—N4—C14—C13	55.54 (16)	C11—C12—C4—N2	−173.92 (13)
C5—N3—C16—C15	146.27 (13)	C11—C12—C4—C3	0.1 (2)
C13—N3—C16—C15	−61.22 (15)	C5—N2—C4—C12	−142.24 (15)
C4—N2—C5—N3	171.00 (13)	C5—N2—C4—C3	44.3 (2)
C4—N2—C5—C1	−4.0 (2)	C9—C3—C4—C12	−2.3 (2)
C13—N3—C5—N2	−5.6 (2)	N1—C3—C4—C12	179.32 (13)
C16—N3—C5—N2	144.38 (14)	C9—C3—C4—N2	171.08 (13)
C13—N3—C5—C1	169.89 (13)	N1—C3—C4—N2	−7.3 (2)
C16—N3—C5—C1	−40.10 (19)	C4—C12—C11—C10	2.5 (2)
C1—C6—C7—C8	179.38 (15)	C9—C10—C11—C12	−2.8 (2)
C1—C6—C7—S1	1.28 (16)	N1—C2—C1—C6	−174.71 (13)
C2—S1—C7—C6	−0.43 (12)	S1—C2—C1—C6	1.34 (16)
C2—S1—C7—C8	−178.71 (13)	N1—C2—C1—C5	5.7 (2)
C5—N3—C13—C14	−146.81 (13)	S1—C2—C1—C5	−178.28 (11)
C16—N3—C13—C14	59.88 (15)	C7—C6—C1—C2	−1.73 (19)
N4—C14—C13—N3	−57.24 (16)	C7—C6—C1—C5	177.87 (13)
C2—N1—C3—C9	126.29 (14)	N2—C5—C1—C2	−38.1 (2)
C2—N1—C3—C4	−55.31 (18)	N3—C5—C1—C2	146.84 (14)
C17—N4—C15—C16	−176.32 (13)	N2—C5—C1—C6	142.37 (16)
C14—N4—C15—C16	−55.47 (16)	N3—C5—C1—C6	−32.7 (2)
N3—C16—C15—N4	58.31 (16)	C11—C10—C9—C3	0.6 (2)
C3—N1—C2—C1	55.2 (2)	C4—C3—C9—C10	2.0 (2)
C3—N1—C2—S1	−120.37 (13)	N1—C3—C9—C10	−179.59 (13)
C7—S1—C2—C1	−0.55 (12)		

### Hydrogen-bond geometry (Å, º)

**Table:**

*D*—H···*A*	*D*—H	H···*A*	*D*···*A*	*D*—H···*A*
N1—H1*N*···O2*S*^i^	0.90 (2)	2.04 (2)	2.933 (2)	177 (2)
O1*S*—H3*S*···N4^ii^	0.86 (2)	1.94 (2)	2.778 (2)	167 (2)

Symmetry codes: (i) −*x*, −*y*+2, −*z*+1; (ii) −*x*+1, −*y*+1, −*z*+1.

## References

[bb1] Allen, F. H., Johnson, O., Shields, G. P., Smith, B. R. & Towler, M. (2004). *J. Appl. Cryst.* **37**, 335–338.

[bb2] Bhardwaj, R. M., Price, L. S., Price, S. L., Reutzel-Edens, S. M., Miller, G. J., Oswald, I. D. H., Johnston, B. & Florence, A. J. (2013). *Cryst. Growth Des.* **3**, 1602–1617.

[bb3] Bruker (2007). *APEX2*, *SAINT* and *SADABS* Bruker AXS Inc., Madison, Wisconsin, USA.

[bb4] Farrugia, L. J. (2012). *J. Appl. Cryst.* **45**, 849–854.

[bb5] Florence, A. J., Baumgartner, B., Weston, C., Shankland, N., Kennedy, A. R., Shankland, K. & David, W. I. F. (2003). *J. Pharm. Sci.* **92**, 1930–1938. 10.1002/jps.10459 12950010

[bb6] Fulton, B. & Goa, K. L. (1997). *Drugs*, **53**, 281–298. 10.2165/00003495-199753020-00007 9028746

[bb7] Gelbrich, T. & Hursthouse, M. B. (2005). *CrystEngComm*, **7**, 324–336.

[bb8] Macrae, C. F., Bruno, I. J., Chisholm, J. A., Edgington, P. R., McCabe, P., Pidcock, E., Rodriguez-Monge, L., Taylor, R., van de Streek, J. & Wood, P. A. (2008). *J. Appl. Cryst.* **41**, 466–470.

[bb9] Reutzel-Edens, S. M., Bush, J. K., Magee, P. A., Stephenson, G. A. & Byrn, S. R. (2003). *Cryst. Growth Des.* **3**, 897–907.

[bb10] Sanger, T. M., Grundy, S. L., Gibson, P. J., Namjoshi, M. A., Greaney, M. G. & Tohen, M. F. (2001). *J. Clin. Psychiatry*, **62**, 273–281. 10.4088/jcp.v62n0410 11379842

[bb11] Sheldrick, G. M. (2008). *Acta Cryst.* A**64**, 112–122. 10.1107/S0108767307043930 18156677

[bb12] Tollefson, G. D., Beasley, C. M., Tran, P. V., Street, J. S., Krueger, J. A., Tamura, R. N., Graffeo, K. A. & Thieme, M. E. (1997). *Am. J. Psychiatry*, **154**, 457–465. 10.1176/ajp.154.4.457 9090331

[bb13] Westrip, S. P. (2010). *J. Appl. Cryst.* **43**, 920–925.

